# Supplementary material for: Interstitial telomeric sequences promote gross chromosomal rearrangement via multiple mechanisms
Source: Proc Natl Acad Sci U S A. 2024 Nov 27;121(49):e2407314121. doi: 10.1073/pnas.2407314121 (PMC11626172; doi:10.1073/pnas.2407314121)
Supplement: Supplementary file 1 — Appendix 01 (PDF) [file pnas.2407314121.sapp.pdf]

Table S1. GCR rate increases with ITS length.

Insertion Length (bp)	Wild-type ITS	*tlc1-tm* ITS	Reverse ITS	Lambda phage DNA	Wild-type ITS; *sir2Δ*
0	$1.6x10^{-9}$ (1)				
18	$3.9x10^{-9}$ (2.4)	$2.4x10^{-9}$ (1.5)	ND	ND	ND
34	$2.2x10^{-7}$ (136)	$1.8x10^{-8}$ (11)	ND	ND	ND
50	$4.5x10^{-6}$ (2749)	$1.4x10^{-7}$ (87)	$5.0x10^{-9}$ (3)	ND	ND
100	$7.2x10^{-6}$ (4414)	$2.1x10^{-6}$ (1278)	$1.3x10^{-7}$ (77)	ND	ND
200	$4.2x10^{-5}$ (25,533)	$2.3x10^{-6}$ (1431)	ND	ND	ND
300	$1.5x10^{-4}$ (89,709)	$5.2x10^{-6}$ (3176)	$1.7x10^{-6}$ (1025)	$1.4x10^{-9}$	$1.9x10^{-4}$

Numbers in parentheses indicate the fold change relative to the no-ITS control. ND: not determined.

Table S2. GCR rates of strains with different 50-bp insertions.

50-bp DNA sequence	GCR rate
Wild-type $(TG)_{0\text{-}6}TGGGTGTG(G)_{0\text{-}1}$ repeats	$4.5x10^{-6}$ (2749)
tlc1-tm $[(TG)_{0\text{-}4}TGG]_nATTTGG$ repeats	$1.4x10^{-7}$ (87)
$(TG)_n$	$4.8x10^{-7}$ (293)
$(TTGGGG)_n$	$5.2x10^{-8}$ (32)
$(GGGGCC)_n$	$4.5x10^{-9}$ (2.8)
$(GGCCCC)_n$	$2.3x10^{-9}$ (1.4)
$(CCCTCT)_n$	$5.1x10^{-9}$ (3.1)
$(AGAGGG)_n$	$4.7x10^{-9}$ (2.8)

Numbers in parentheses indicate the fold change relative to the no-ITS control.

Table S3. GCR rates of mutants identified in the ITS-GCR screen without and with a 50-bp ITS.

Genotype	No ITS	50-bp ITS
30°C		
Wild type	$3.5x10^{-10}$ (1)[a]	$4.5x10^{-6}$ (1)
elg1Δ	$1.6x10^{-7}$ (45)[a]	$1.4x10^{-5}$ (3.0)
rad27Δ	$3.6x10^{-7}$ (1040)[a]	$1.6x10^{-4}$ (36)
ydl162cΔ	$4.6x10^{-9}$ (13)[a]	$3.4x10^{-5}$ (7.6)
22°C		
Wild type	$1.9x10^{-9}$ (1)[b]	$2.3x10^{-6}$ (1)
cdc2-2	$1.4x10^{-8}$ (7.5)[b]	$1.1x10^{-5}$ (4.7)
cdc2-7	$2.3x10^{-8}$ (12)[b]	$1.2x10^{-5}$ (5.3)
cdc9-1	$5.4x10^{-7}$ (283)	$1.2x10^{-5}$ (5.5)
rfc2-1	$6.8x10^{-9}$ (3.5)[b]	$7.4x10^{-6}$ (3.3)
rfc4-20	$4.0x10^{-7}$ (209)[b]	$2.1x10^{-5}$ (9.3)
rfc5-1	$8.7x10^{-9}$ (4.5)[b]	$5.9x10^{-6}$ (2.6)
rpt4-150	$1.6x10^{-9}$ (0.9)[b]	$1.2x10^{-5}$ (5.5)

Numbers in parentheses indicate the fold change relative to the corresponding wild-type control.
[a]Data taken from a previous publication using the original GCR assay without an ITS (1).
[b]These values were derived from strains without an ITS, but with the *hphMX* marker inserted at the *PRB1* locus.

Table S4. GCR rates of selected mutants not identified in the ITS-GCR screen without and with a 50-bp ITS.

Genotype	No ITS[a]	50-bp ITS
Wild type	$3.5x10^{-10}$ (1)	$4.5x10^{-6}$ (1)
pif1Δ	$3.5x10^{-7}$ (1010)	$1.1x10^{-5}$ (2.5)
rad50Δ	$2.3x10^{-7}$ (657)	$3.3x10^{-6}$ (0.72)
sic1Δ	$1.8x10^{-7}$ (514)	$4.5x10^{-6}$ (1.0)
rlf2Δ	$1.2x10^{-7}$ (343)	$4.0x10^{-6}$ (0.9)
rmi1Δ	$6.0x10^{-8}$ (189)	$7.4x10^{-6}$ (1.6)
mus81Δ	$6.5x10^{-8}$ (186)	$4.5x10^{-6}$ (1.0)
rad52Δ	$4.0x10^{-8}$ (115)	$5.4x10^{-5}$ (12.1)
slx8Δ	$2.6x10^{-8}$ (75)	$6.8x10^{-6}$ (1.5)
slx5Δ	$2.3x10^{-8}$ (66)	$1.2x10^{-5}$ (2.7)
sgs1Δ	$1.2x10^{-8}$ (34)	$3.7x10^{-6}$ (0.81)
rrm3Δ	$1.4x10^{-9}$ (4.0)	$1.7x10^{-5}$ (3.8)
rad51Δ	$3.5x10^{-9}$ (10)	$5.5x10^{-6}$ (1.2)
pol32Δ	$4.0x10^{-10}$ (1.1)	$2.5x10^{-6}$ (0.5)

Numbers in parentheses indicate the fold change relative to the wild-type control.
[a]Data taken from a previous publication using the original GCR assay without an ITS (1).

Table S5. List of mutants identified in the screen that decrease ITS-induced GCRs.

Mutant	% of GCR colonies	Mutant	% of GCR colonies	Mutant	% of GCR colonies
abd1-8	0	*ies6Δ*	0	*sgv1-23*	0
abf1-103	0	*ilv1Δ*	21	*sgv1-35*	8
act1-105	0	*ino1Δ*	13	*she4Δ*	8
ado1Δ	0	*ipk1Δ*	17	*sit4Δ*	0
anp1Δ	22	*lcb1-10*	0	*sla1Δ*	13
arp7-E411K	29	*ldb7Δ*	0	*slu7-ts2*	0
arv1Δ	0	*ldb16Δ*	22	*smc4-1*	2
atp11Δ	0	*leo1Δ*	8 ($3.7x10^{-7}$)	*smt3-331*	9
bbp1-1	0	*lip2Δ*	38	*snf2Δ*	6
bdf1Δ	0	*lpd1Δ*	38	*snf4Δ*	32
bfr1Δ	0	*mct1Δ*	17	*snf8Δ*	4
brl1-C371S	5	*mdj1Δ*	22	*spn1-K192N*	17
bro1Δ	8	*mog1Δ*	0	*spt3Δ*	0
bud25Δ	8	*mot1-1033*	9	*spt4Δ*	0 ($3.6x10^{-7}$)
cdc5-1	0	*mps1-3796*	16	*spt10Δ*	8
cdc8-2	29	*mps2-1*	5	*spt15-I143N*	4
cdc13-1	25 ($8.3x10^{-7}$)	*mps3-1*	13	*ssn3Δ*	0
cdc24-5	16	*mss4-102*	8	*ssq1Δ*	0
cdc25-1	0	*mtr4-1*	0	*stu2-10*	0
cdc36-16	5	*ndc1-4*	13	*swc4-4*	0
cdc55Δ	17	*ngg1Δ*	30	*swc5Δ*	26
ceg1-3	0	*npl6Δ*	0	*swi4Δ*	21
ceg1-C354	0	*nup159-1*	0	*taf2-1*	12
chc1Δ	0	*opi3Δ*	33	*tfb5Δ*	0 ($4.5x10^{-7}$)

cks1-35	0	*pdx3Δ*	4	*trk1Δ*	0
cox6Δ	13	*pmp3Δ*	25	*trm1Δ*	0
cox15Δ	25	*poc4Δ*	0	*trm10Δ*	0
cse2Δ	23	*pol1-17*	0	*tsc3-2*	0
ctf8-162	5	*por1Δ*	0	*vam10Δ*	33
ctk2Δ	9 (2.7×10^{-7})	*prp9-ts*	8	*vma3Δ*	35
ctk3Δ	8	*prp18-ts*	21	*vma8Δ*	16
dbp5-1	0	*prp31-ts*	0	*vma21Δ*	29
dbp7Δ	33	*prs2Δ*	0	*vps3Δ*	13
def1Δ	0	*prs3Δ*	0	*vps5Δ*	33
dfr1-td	16	*rad1Δ*	17 (7.9×10^{-7})	*vps16Δ*	4
dhh1Δ	0	*rad2Δ*	21 (1.4×10^{-6})	*vps36Δ*	4
dna2-1	13	*rad4Δ*	8 (1.1×10^{-6})	*vps64Δ*	6
dsl1-DC30	0	*rad10Δ*	17 (1.6×10^{-6})	*vps69Δ*	0
erg2Δ	33	*rad14Δ*	13 (1.3×10^{-6})	*vps71Δ*	17
erg3Δ	33	*rad23Δ*	38 (1.7×10^{-6})	*yaf9Δ*	8
est1Δ	0 (2.6×10^{-8})	*rad33Δ*	17 (1.9×10^{-6})	*ybl100cΔ*	25
est2Δ	0 (2.2×10^{-8})	*rap1-1*	4 (2.9×10^{-8})	*ycs4-1*	24
est3Δ	24 (2.1×10^{-8})	*ref2Δ*	0	*ydl118wΔ*	17
gcv3Δ	17	*rio2-1*	18	*ydr417cΔ*	4
get1Δ	5	*rpc40-W78R*	0	*yef3-F650S*	17
get2Δ	0.0	*rpl8aΔ*	13	*yel045cΔ*	0
glc7-10	5	*rpl12bΔ*	20	*ygl072cΔ*	9
gpa1-ts	6	*rpl21bΔ*	4	*ygl188c-aΔ*	8
gpm2Δ	5	*rpl34bΔ*	4	*ygl214wΔ*	13
grr1Δ	25	*rpp1aΔ*	17	*yhc1-2*	0
grx5Δ	0	*rsc1Δ*	9	*yjl175wΔ*	33
gsh1Δ	33	*rsc2Δ*	17	*yjr039wΔ*	25
hnt3Δ	17	*rse1-1*	0	*ykl096c-bΔ*	0
hoc1Δ	0	*rtf1Δ*	13 (5.0×10^{-7})	*ylr358cΔ*	25
hop2Δ	25	*sac1Δ*	13	*yml013c-aΔ*	0
hpr1Δ	0	*sac3Δ*	25	*yml096wΔ*	38
htz1Δ	38	*scd5-D338*	0	*ynl296wΔ*	4
hyp2-2	22	*scd5-PP1D2*	0	*yor200wΔ*	33
ice2Δ	21	*sfi1-7*	4	*zwf1Δ*	13
ies2Δ	4	*sfp1Δ*	33		

YKO and ts strains were tested at 30°C and 22°C, respectively. "% of GCR colonies" refers to the percentage of colonies that were able to grow on selective (canavanine and 5-FOA) media in the high-throughput screen. For comparison, the median percentage was 79% for strains in the YKO library and 67% for strains in the ts library. GCR rates (in parentheses) were obtained with fluctuation tests for some of the strains. The GCR rate for the wild-type strain without an ITS (ZYY139) is 4.4×10^{-9} at 30°C and 5.6×10^{-9} at 22°C. The GCR rate for the wild-type strain with a 50-bp ITS (ZYY141) is 7.5×10^{-6} at 30°C and 4.5×10^{-6} at 22°C.

Table S6. GCR rates of telomere and NER mutants with a 50-bp ITS.

Genotype	GCR rate
30°C	
Wild type without ITS	$4.4x10^{-9}$ (1)
Wild type	$7.5x10^{-6}$ (1720)
est1Δ	$2.5x10^{-8}$ (5.8)
est2Δ	$2.2x10^{-8}$ (5.0)
est3Δ	$2.1x10^{-8}$ (4.8)
rad4Δ	$1.1x10^{-6}$ (262)
rad23Δ	$1.7x10^{-6}$ (389)
rad33Δ	$1.9x10^{-6}$ (428)
rad14Δ	$1.3x10^{-6}$ (301)
rad1Δ	$9.8x10^{-7}$ (225)
rad10Δ	$1.6x10^{-6}$ (362)
rad2Δ	$1.4x10^{-6}$ (315)
tfb5Δ	$4.5x10^{-7}$ (103)
rad7Δ	$6.5x10^{-6}$ (1493)
rad16Δ	$6.2x10^{-6}$ (1419)
rad26Δ	$2.3x10^{-6}$ (530)
22°C	
Wild type without ITS	$5.6x10^{-9}$ (1)
Wild type	$4.5x10^{-6}$ (815)
cdc13-1	$8.3x10^{-7}$ (148)
rap1-1	$2.9x10^{-8}$ (5.1)

Numbers in parentheses indicate the fold change relative to the no-ITS control.
Gene deletions in bold were not identified in the screen.

Table S7. GCR rates of strains lacking RNase H.

Genotype	No ITS	50-bp ITS
Wild type	$4.4x10^{-9}$ (1)	$7.5x10^{-6}$ (1)
rnh1Δ	$6.6x10^{-9}$ (1.5)	$6.8x10^{-6}$ (0.9)
rnh201Δ	$8.7x10^{-9}$ (2.0)	$8.1x10^{-6}$ (1.1)
rnh1Δ rnh201Δ	$6.9x10^{-8}$ (16)	$1.0x10^{-5}$ (1.3)

Table S8. GCR rates of wild-type strains with a 50-bp ITS overexpressing RNase H.

Overexpressed gene	GCR rate
–	$5.9x10^{-6}$ (1)
RNH1	$3.5x10^{-5}$ (6.0)
rnh1-D193N[a]	$6.6x10^{-5}$ (11)

[a]overexpresses catalytically dead Rnh1

Table S9. Yeast strains used in this study.

Strain name	Genotype	Source
FRY806	MATα ADE2 CAN1 his3-11,15 leu2-3,112 lys2Δ trp1-1 ura3-1 RAD5 hxt13ΔURA3 prb1ΔhphMX	This study
FRY871	MATα ADE2 CAN1 his3-11,15 leu2-3,112 lys2Δ trp1-1 ura3-1 RAD5 hxt13ΔURA3 prb1ΔhphMX-18bp_ITS	This study
FRY873	MATα ADE2 CAN1 his3-11,15 leu2-3,112 lys2Δ trp1-1 ura3-1 RAD5 hxt13ΔURA3 prb1ΔhphMX-34bp_ITS	This study
FRY808	MATα ADE2 CAN1 his3-11,15 leu2-3,112 lys2Δ trp1-1 ura3-1 RAD5 hxt13ΔURA3 prb1ΔhphMX-50bp_ITS	This study
FRY879	MATα ADE2 CAN1 his3-11,15 leu2-3,112 lys2Δ trp1-1 ura3-1 RAD5 hxt13ΔURA3 prb1ΔhphMX-100bp_ITS	This study
JBY9	MATα ADE2 CAN1 his3-11,15 leu2-3,112 lys2Δ trp1-1 ura3-1 RAD5 hxt13ΔURA3 prb1ΔhphMX-200bp_ITS	This study
FRY810	MATα ADE2 CAN1 his3-11,15 leu2-3,112 lys2Δ trp1-1 ura3-1 RAD5 hxt13ΔURA3 prb1ΔhphMX-300bp_ITS	This study
FRY875	MATα ADE2 CAN1 his3-11,15 leu2-3,112 lys2Δ trp1-1 ura3-1 RAD5 hxt13ΔURA3 prb1ΔhphMX-18bp_tlc1-tm_ITS	This study
FRY877	MATα ADE2 CAN1 his3-11,15 leu2-3,112 lys2Δ trp1-1 ura3-1 RAD5 hxt13ΔURA3 prb1ΔhphMX-34bp_tlc1-tm_ITS	This study
FRY812	MATα ADE2 CAN1 his3-11,15 leu2-3,112 lys2Δ trp1-1 ura3-1 RAD5 hxt13ΔURA3 prb1ΔhphMX-50bp_tlc1-tm_ITS	This study
FRY881	MATα ADE2 CAN1 his3-11,15 leu2-3,112 lys2Δ trp1-1 ura3-1 RAD5 hxt13ΔURA3 prb1ΔhphMX-100bp_tlc1-tm_ITS	This study
JBY10	MATα ADE2 CAN1 his3-11,15 leu2-3,112 lys2Δ trp1-1 ura3-1 RAD5 hxt13ΔURA3 prb1ΔhphMX-200bp_tlc1-tm_ITS	This study
FRY814	MATα ADE2 CAN1 his3-11,15 leu2-3,112 lys2Δ trp1-1 ura3-1 RAD5 hxt13ΔURA3 prb1ΔhphMX-300bp_tlc1-tm_ITS	This study
ZYY439	MATα ADE2 CAN1 his3-11,15 leu2-3,112 lys2Δ trp1-1 ura3-1 RAD5 hxt13ΔURA3 prb1ΔhphMX-50bp_revITS	This study
ZYY445	MATα ADE2 CAN1 his3-11,15 leu2-3,112 lys2Δ trp1-1 ura3-1 RAD5 hxt13ΔURA3 prb1ΔhphMX-100bp_revITS	This study
FRY816	MATα ADE2 CAN1 his3-11,15 leu2-3,112 lys2Δ trp1-1 ura3-1 RAD5 hxt13ΔURA3 prb1ΔhphMX-300bp_revITS	This study
FRY818	MATα ADE2 CAN1 his3-11,15 leu2-3,112 lys2Δ trp1-1 ura3-1 RAD5 hxt13ΔURA3 prb1ΔhphMX-300bp_λDNA	This study
JBY1	MATα ADE2 CAN1 his3-11,15 leu2-3,112 lys2Δ trp1-1 ura3-1 RAD5 hxt13ΔURA3 prb1ΔhphMX-300bp_ITS sir2ΔnatMX	This study
SOY73	MATα ADE2 CAN1 his3-11,15 leu2-3,112 lys2Δ trp1-1 ura3-1 RAD5 hxt13ΔURA3 prb1ΔhphMX-$(TG)_{25}$	This study
FRY1110	MATα ADE2 CAN1 his3-11,15 leu2-3,112 lys2Δ trp1-1 ura3-1 RAD5 hxt13ΔURA3 prb1ΔhphMX-50bp_G_4T_2	This study
SOY19	MATα ADE2 CAN1 his3-11,15 leu2-3,112 lys2Δ trp1-1 ura3-1 RAD5 hxt13ΔURA3 prb1ΔhphMX-50bp_G_4C_2	This study
SOY20	MATα ADE2 CAN1 his3-11,15 leu2-3,112 lys2Δ trp1-1 ura3-1 RAD5 hxt13ΔURA3 prb1ΔhphMX-50bp_C_4G_2	This study
SOY30	MATα ADE2 CAN1 his3-11,15 leu2-3,112 lys2Δ trp1-1 ura3-1 RAD5 hxt13ΔURA3 prb1ΔhphMX-50bp_AGAGGG	This study
SOY29	MATα ADE2 CAN1 his3-11,15 leu2-3,112 lys2Δ trp1-1 ura3-1 RAD5 hxt13ΔURA3 prb1ΔhphMX-50bp_CCCTCT	This study
DDY4600	MAT**a**-inc ura3-52 lys2-801 ade2-101 ochre trp1-Δ63 his3-Δ200 leu2-Δ1::P_{GAL1}-HO-LEU2 rad52::HIS3 VII-L::TG82-HOcs-LYS2 ura3::hphMX cdc13::kanMX + pRS425-CDC13	(2)

FRY820	*MATα his3Δ1 ura3Δ0 leu2Δ0 met15Δ0 lyp1Δ hxt13ΔURA3 mfa1::*P_{MFA1}*-HIS3 prb1ΔhphMX*	This study
FRY910	*MATα his3Δ1 ura3Δ0 leu2Δ0 met15Δ0 lyp1Δ hxt13ΔURA3 mfa1::*P_{MFA1}*-HIS3 prb1ΔhphMX-50bp_ITS*	This study
ZYY114	*MAT***a** *ura3Δ0 leu2Δ0 met15Δ0 lyp1Δ hxt13ΔURA3 mfa1::*P_{MFA1}*-HIS3 prb1ΔhphMX-50bp_ITS his3ΔkanMX*	This study
YYY24	*MAT***a** *his3Δ1 ura3Δ0 leu2Δ0 met15Δ0 lyp1Δ hxt13ΔURA3 mfa1::*P_{MFA1}*-HIS3 prb1ΔhphMX-50bp_ITS bub2ΔkanMX*	This study
FRY1045	*MATα ADE2 CAN1 his3-11,15 leu2-3,112 lys2Δ trp1-1 ura3-1 RAD5 hxt13ΔURA3 prb1ΔhphMX-50bp_ITS cdc2-2::kanMX*	This study
FRY1030	*MATα ADE2 CAN1 his3-11,15 leu2-3,112 lys2Δ trp1-1 ura3-1 RAD5 hxt13ΔURA3 prb1ΔhphMX cdc2-2::kanMX*	This study
FRY1046	*MATα ADE2 CAN1 his3-11,15 leu2-3,112 lys2Δ trp1-1 ura3-1 RAD5 hxt13ΔURA3 prb1ΔhphMX-50bp_ITS cdc2-7::kanMX*	This study
FRY1032	*MATα ADE2 CAN1 his3-11,15 leu2-3,112 lys2Δ trp1-1 ura3-1 RAD5 hxt13ΔURA3 prb1ΔhphMX cdc2-7::kanMX*	This study
FRY1054	*MATα ADE2 CAN1 his3-11,15 leu2-3,112 lys2Δ trp1-1 ura3-1 RAD5 hxt13ΔURA3 prb1ΔhphMX-50bp_ITS cdc9-1::kanMX*	This study
FRY1041	*MATα ADE2 CAN1 his3-11,15 leu2-3,112 lys2Δ trp1-1 ura3-1 RAD5 hxt13ΔURA3 prb1ΔhphMX cdc9-1::kanMX*	This study
ZYY19	*MATα ADE2 CAN1 his3-11,15 leu2-3,112 lys2Δ trp1-1 ura3-1 RAD5 hxt13ΔURA3 prb1ΔhphMX-50bp_ITS elg1ΔkanMX*	This study
ZYY8	*MATα ADE2 CAN1 his3-11,15 leu2-3,112 lys2Δ trp1-1 ura3-1 RAD5 hxt13ΔURA3 prb1ΔhphMX-50bp_ITS rad27ΔkanMX*	This study
FRY1051	*MATα ADE2 CAN1 his3-11,15 leu2-3,112 lys2Δ trp1-1 ura3-1 RAD5 hxt13ΔURA3 prb1ΔhphMX-50bp_ITS rfc2-1::kanMX*	This study
FRY1038	*MATα ADE2 CAN1 his3-11,15 leu2-3,112 lys2Δ trp1-1 ura3-1 RAD5 hxt13ΔURA3 prb1ΔhphMX rfc2-1::kanMX*	This study
FRY1053	*MATα ADE2 CAN1 his3-11,15 leu2-3,112 lys2Δ trp1-1 ura3-1 RAD5 hxt13ΔURA3 prb1ΔhphMX-50bp_ITS rfc4-20::kanMX*	This study
FRY1040	*MATα ADE2 CAN1 his3-11,15 leu2-3,112 lys2Δ trp1-1 ura3-1 RAD5 hxt13ΔURA3 prb1ΔhphMX rfc4-20::kanMX*	This study
FRY1146	*MATα ADE2 CAN1 his3-11,15 leu2-3,112 lys2Δ trp1-1 ura3-1 RAD5 hxt13ΔURA3 prb1ΔhphMX-50bp_ITS rfc5-1::kanMX*	This study
FRY1144	*MATα ADE2 CAN1 his3-11,15 leu2-3,112 lys2Δ trp1-1 ura3-1 RAD5 hxt13ΔURA3 prb1ΔhphMX rfc5-1::kanMX*	This study
FRY1150	*MATα ADE2 CAN1 his3-11,15 leu2-3,112 lys2Δ trp1-1 ura3-1 RAD5 hxt13ΔURA3 prb1ΔhphMX-50bp_ITS rpt4-150::kanMX*	This study
FRY1148	*MATα ADE2 CAN1 his3-11,15 leu2-3,112 lys2Δ trp1-1 ura3-1 RAD5 hxt13ΔURA3 prb1ΔhphMX rpt4-150::kanMX*	This study
FRY1025	*MATα ADE2 CAN1 his3-11,15 leu2-3,112 lys2Δ trp1-1 ura3-1 RAD5 hxt13ΔURA3 prb1ΔhphMX-50bp_ITS ydl162cΔkanMX*	This study
FRY1100	*MATα ADE2 CAN1 his3-11,15 leu2-3,112 lys2Δ trp1-1 ura3-1 RAD5 hxt13ΔURA3 prb1ΔhphMX-50bp_ITS pif1ΔkanMX*	This study
FRY1108	*MATα ADE2 CAN1 his3-11,15 leu2-3,112 lys2Δ trp1-1 ura3-1 RAD5 hxt13ΔURA3 prb1ΔhphMX-50bp_ITS rad50ΔkanMX*	This study
FRY1114	*MATα ADE2 CAN1 his3-11,15 leu2-3,112 lys2Δ trp1-1 ura3-1 RAD5 hxt13ΔURA3 prb1ΔhphMX-50bp_ITS sic1ΔkanMX*	This study
ZYY307	*MATα ADE2 CAN1 his3-11,15 leu2-3,112 lys2Δ trp1-1 ura3-1 RAD5 hxt13ΔURA3 prb1ΔhphMX-50bp_ITS rlf2ΔkanMX*	This study
FRY1120	*MATα ADE2 CAN1 his3-11,15 leu2-3,112 lys2Δ trp1-1 ura3-1 RAD5 hxt13ΔURA3 prb1ΔhphMX-50bp_ITS rmi1ΔkanMX*	This study
ZYY321	*MATα ADE2 CAN1 his3-11,15 leu2-3,112 lys2Δ trp1-1 ura3-1 RAD5 hxt13ΔURA3 prb1ΔhphMX-50bp_ITS mus81ΔkanMX*	This study

FRY1126	MATα ADE2 CAN1 his3-11,15 leu2-3,112 lys2Δ trp1-1 ura3-1 RAD5 hxt13ΔURA3 prb1ΔhphMX-50bp_ITS rad52ΔkanMX	This study
FRY1130	MATα ADE2 CAN1 his3-11,15 leu2-3,112 lys2Δ trp1-1 ura3-1 RAD5 hxt13ΔURA3 prb1ΔhphMX-50bp_ITS slx8ΔkanMX	This study
FRY1136	MATα ADE2 CAN1 his3-11,15 leu2-3,112 lys2Δ trp1-1 ura3-1 RAD5 hxt13ΔURA3 prb1ΔhphMX-50bp_ITS slx5ΔkanMX	This study
FRY1140	MATα ADE2 CAN1 his3-11,15 leu2-3,112 lys2Δ trp1-1 ura3-1 RAD5 hxt13ΔURA3 prb1ΔhphMX-50bp_ITS sgs1ΔkanMX	This study
ZYY61	MATα ADE2 CAN1 his3-11,15 leu2-3,112 lys2Δ trp1-1 ura3-1 RAD5 hxt13ΔURA3 prb1ΔhphMX-50bp_ITS rrm3ΔkanMX	This study
ZYY417	MATα ADE2 CAN1 his3-11,15 leu2-3,112 lys2Δ trp1-1 ura3-1 RAD5 hxt13ΔURA3 prb1ΔhphMX-50bp_ITS rad51ΔkanMX	This study
ZYY421	MATα ADE2 CAN1 his3-11,15 leu2-3,112 lys2Δ trp1-1 ura3-1 RAD5 hxt13ΔURA3 prb1ΔhphMX-50bp_ITS pol32ΔkanMX	This study
ZYY238	MAT**a**/α ade2-1/ade2-1 can1-100/can1-100 his3-11,15/his3-11,15 leu2-3,112/leu2-3,112 trp1-1/trp1-1 ura3-1/ura3-1 RAD5/RAD5 est2ΔURA3/EST2 cdc2-7::kanMX/CDC2	This study
ZYY236	MAT**a**/α ade2-1/ade2-1 can1-100/can1-100 his3-11,15/his3-11,15 leu2-3,112/leu2-3,112 trp1-1/trp1-1 ura3-1/ura3-1 RAD5/RAD5 est2ΔURA3/EST2 cdc9-1::kanMX/CDC9	This study
ZYY232	MAT**a**/α ade2-1/ade2-1 can1-100/can1-100 his3-11,15/his3-11,15 leu2-3,112/leu2-3,112 trp1-1/trp1-1 ura3-1/ura3-1 RAD5/RAD5 est2ΔURA3/EST2 elg1ΔkanMX/ELG1	This study
ZYY244	MAT**a**/α ade2-1/ade2-1 can1-100/can1-100 his3-11,15/his3-11,15 leu2-3,112/leu2-3,112 trp1-1/trp1-1 ura3-1/ura3-1 RAD5/RAD5 est2ΔURA3/EST2 rfc2-1::kanMX/RFC2	This study
ZYY246	MAT**a**/α ade2-1/ade2-1 can1-100/can1-100 his3-11,15/his3-11,15 leu2-3,112/leu2-3,112 trp1-1/trp1-1 ura3-1/ura3-1 RAD5/RAD5 est2ΔURA3/EST2 rfc4-20::kanMX/RFC4	This study
ZYY248	MAT**a**/α ade2-1/ade2-1 can1-100/can1-100 his3-11,15/his3-11,15 leu2-3,112/leu2-3,112 trp1-1/trp1-1 ura3-1/ura3-1 RAD5/RAD5 est2ΔURA3/EST2 rfc5-1::kanMX/RFC5	This study
ZYY240	MAT**a**/α ade2-1/ade2-1 can1-100/can1-100 his3-11,15/his3-11,15 leu2-3,112/leu2-3,112 trp1-1/trp1-1 ura3-1/ura3-1 RAD5/RAD5 est2ΔURA3/EST2 rpt4-150::kanMX/RPT4	This study
ZYY228	MAT**a**/α ade2-1/ade2-1 can1-100/can1-100 his3-11,15/his3-11,15 leu2-3,112/leu2-3,112 trp1-1/trp1-1 ura3-1/ura3-1 RAD5/RAD5 est2ΔURA3/EST2 ydl162cΔkanMX/YDL162C	This study
DNY194	MATα his3Δ1 ura3Δ0 leu2Δ0 met15Δ0 lyp1Δ hxt13ΔURA3 mfa1::P_{MFA1}-HIS3 ho::CIN8-natMX prb1ΔhphMX-50bp_ITS	This study
ZYY162	MAT**a** his3Δ1 ura3Δ0 leu2Δ0 met15Δ0 lyp1Δ hxt13ΔURA3 mfa1::P_{MFA1}-HIS3 ho::CIN8-natMX prb1ΔhphMX	This study
ZYY178	MAT**a** ura3Δ0 leu2Δ0 met15Δ0 lyp1Δ hxt13ΔURA3 mfa1::P_{MFA1}-HIS3 ho::CIN8-natMX prb1ΔhphMX-50bp_ITS his3ΔkanMX	This study
ZYY261	MATα his3Δ1 ura3Δ0 leu2Δ0 met15Δ0 lyp1Δ mfa1::MFA1pr-HIS3 prb1Δ::hphMX (de novo telomere added after hphMX)	This study
YYY69	MAT**a** his3Δ1 ura3Δ0 leu2Δ0 met15Δ0 lyp1Δ hxt13ΔURA3 mfa1::P_{MFA1}-HIS3 prb1ΔhphMX-50bp_ITS rad4ΔkanMX	This study
YYY65	MAT**a** his3Δ1 ura3Δ0 leu2Δ0 met15Δ0 lyp1Δ hxt13ΔURA3 mfa1::P_{MFA1}-HIS3 prb1ΔhphMX-50bp_ITS rad23ΔkanMX	This study
YYY67	MAT**a** his3Δ1 ura3Δ0 leu2Δ0 met15Δ0 lyp1Δ hxt13ΔURA3 mfa1::P_{MFA1}-HIS3 prb1ΔhphMX-50bp_ITS rad33ΔkanMX	This study
YYY63	MAT**a** his3Δ1 ura3Δ0 leu2Δ0 met15Δ0 lyp1Δ hxt13ΔURA3 mfa1::P_{MFA1}-HIS3 prb1ΔhphMX-50bp_ITS rad14ΔkanMX	This study

YYY57	MAT**a** his3Δ1 ura3Δ0 leu2Δ0 met15Δ0 lyp1Δ hxt13ΔURA3 mfa1::P_{MFA1}-HIS3 prb1ΔhphMX-50bp_ITS rad1ΔkanMX	This study
ZYY224	MAT**a** his3Δ1 ura3Δ0 leu2Δ0 met15Δ0 lyp1Δ hxt13ΔURA3 mfa1::P_{MFA1}-HIS3 prb1ΔhphMX-50bp_ITS rad1ΔkanMX	This study
YYY61	MAT**a** his3Δ1 ura3Δ0 leu2Δ0 met15Δ0 lyp1Δ hxt13ΔURA3 mfa1::P_{MFA1}-HIS3 prb1ΔhphMX-50bp_ITS rad10ΔkanMX	This study
YYY59	MAT**a** his3Δ1 ura3Δ0 leu2Δ0 met15Δ0 lyp1Δ hxt13ΔURA3 mfa1::P_{MFA1}-HIS3 prb1ΔhphMX-50bp_ITS rad2ΔkanMX	This study
ZYY186	MAT**a** his3Δ1 ura3Δ0 leu2Δ0 met15Δ0 lyp1Δ hxt13ΔURA3 mfa1::P_{MFA1}-HIS3 prb1ΔhphMX-50bp_ITS tfb5ΔkanMX	This study
ZYY315	MAT**a** his3Δ1 ura3Δ0 leu2Δ0 met15Δ0 lyp1Δ hxt13ΔURA3 mfa1::P_{MFA1}-HIS3 prb1ΔhphMX-50bp_ITS cdc13-1::kanMX	This study
YYY102	MAT**a** his3Δ1 ura3Δ0 leu2Δ0 met15Δ0 lyp1Δ hxt13ΔURA3 mfa1::P_{MFA1}-HIS3 prb1ΔhphMX-50bp_ITS ctk2ΔkanMX	This study
YYY84	MAT**a** his3Δ1 ura3Δ0 leu2Δ0 met15Δ0 lyp1Δ hxt13ΔURA3 mfa1::P_{MFA1}-HIS3 prb1ΔhphMX-50bp_ITS leo1ΔkanMX	This study
ZYY317	MAT**a** his3Δ1 ura3Δ0 leu2Δ0 met15Δ0 lyp1Δ hxt13ΔURA3 mfa1::P_{MFA1}-HIS3 prb1ΔhphMX-50bp_ITS rap1-1::kanMX	This study
YYY86	MAT**a** his3Δ1 ura3Δ0 leu2Δ0 met15Δ0 lyp1Δ hxt13ΔURA3 mfa1::P_{MFA1}-HIS3 prb1ΔhphMX-50bp_ITS rtf1ΔkanMX	This study
ZYY184	MAT**a** his3Δ1 ura3Δ0 leu2Δ0 met15Δ0 lyp1Δ hxt13ΔURA3 mfa1::P_{MFA1}-HIS3 prb1ΔhphMX-50bp_ITS spt4ΔkanMX	This study
ZYY141	MAT**a** his3Δ1 ura3Δ0 leu2Δ0 met15Δ0 lyp1Δ hxt13ΔURA3 mfa1::P_{MFA1}-HIS3 prb1ΔhphMX-50bp_ITS	This study
ZYY301	MAT**a** his3Δ1 ura3Δ0 leu2Δ0 met15Δ0 lyp1Δ hxt13ΔURA3 mfa1::P_{MFA1}-HIS3 prb1ΔhphMX-50bp_ITS rad7ΔkanMX	This study
ZYY303	MATa his3Δ1 ura3Δ0 leu2Δ0 met15Δ0 lyp1Δ hxt13ΔURA3 mfa1::P_{MFA1}-HIS3 prb1ΔhphMX-50bp_ITS rad16ΔkanMX	This study
ZYY309	MAT**a** his3Δ1 ura3Δ0 leu2Δ0 met15Δ0 lyp1Δ hxt13ΔURA3 mfa1::P_{MFA1}-HIS3 prb1ΔhphMX-50bp_ITS rad26ΔkanMX	This study
ZYY407	MAT**a** his3Δ1 ura3Δ0 leu2Δ0 met15Δ0 lyp1Δ hxt13ΔURA3 mfa1::P_{MFA1}-HIS3 prb1ΔhphMX rnh1ΔkanMX	This study
ZYY409	MAT**a** his3Δ1 ura3Δ0 leu2Δ0 met15Δ0 lyp1Δ hxt13ΔURA3 mfa1::P_{MFA1}-HIS3 prb1ΔhphMX-50bp_ITS rnh1ΔkanMX	This study
ZYY411	MAT**a** his3Δ1 ura3Δ0 leu2Δ0 met15Δ0 lyp1Δ hxt13ΔURA3 mfa1::P_{MFA1}-HIS3 prb1ΔhphMX rnh201ΔkanMX	This study
ZYY413	MAT**a** his3Δ1 ura3Δ0 leu2Δ0 met15Δ0 lyp1Δ hxt13ΔURA3 mfa1::P_{MFA1}-HIS3 prb1ΔhphMX-50bp_ITS rnh201ΔkanMX	This study
ZYY423	MAT**a** his3Δ1 ura3Δ0 leu2Δ0 met15Δ0 lyp1Δ hxt13ΔURA3 mfa1::P_{MFA1}-HIS3 prb1ΔhphMX rnh1ΔkanMX rnh201ΔnatMX	This study
ZYY425	MAT**a** his3Δ1 ura3Δ0 leu2Δ0 met15Δ0 lyp1Δ hxt13ΔURA3 mfa1::P_{MFA1}-HIS3 prb1ΔhphMX-50bp_ITS rnh1ΔkanMX rnh201ΔnatMX	This study
ZYY279	MATα ADE2 CAN1 his3-11,15 leu2-3,112 lys2Δ trp1-1 ura3-1 RAD5 hxt13ΔURA3 prb1ΔhphMX-50bp_ITS + pBL190 (pRS423, P_{GPD1})	This study
ZYY281	MATα ADE2 CAN1 his3-11,15 leu2-3,112 lys2Δ trp1-1 ura3-1 RAD5 hxt13ΔURA3 prb1ΔhphMX-50bp_ITS + pBL192 (pRS423, P_{GPD1}-RNH1-HA)	This study
ZYY283	MATα ADE2 CAN1 his3-11,15 leu2-3,112 lys2Δ trp1-1 ura3-1 RAD5 hxt13ΔURA3 prb1ΔhphMX-50bp_ITS + pBL710 (pRS423, P_{GPD1}-rnh1-D193N-HA)	This study

Table S10. ITS and other inserted DNA sequences

**Inserted DNA**	**DNA sequence**
18-bp ITS	TGTGTGGGTGTGGGTGTG
34-bp ITS	GTGTGTGGGTGTGGTGTGTGTGGGTGTGGGTGTG
50-bp ITS	TGTGGGTGTGGTGTGGGTGTGTGGGTGTGGTGTGTGTGGGTGTGGGTGTG
100-bp ITS	GGTGTGGTGTGTGGGTGTGTGGGTGTGGTGTGTGTGTGGGTGTGTGGGTGTGGTGTGGGT<br>GTGGGTGTGGGTGTGGTGTGTGGGTGTGGTGTGGGTGTGG
200-bp ITS	GTGTGGGTGTGGTGTGTGGGTGTGGGTGTGTGGGTGTGGTGTGTGGGTGTGGGTGTGGTG<br>TGGGTGTGTGGGTGTGGTGTGTGTGGGTGTGGTGTGTGTGGGTGTGGTGTGTGGGTGTGT<br>GGGTGTGGTGTGTGTGTGGGTGTGTGGGTGTGGTGTGGGTGTGGGTGTGGGTGTGGTGTG<br>TGGGTGTGGTGTGGGTGTGG
300-bp ITS	TGTGGGTGTGGTGTGGGTGTGTGGGTGTGGTGTGTGTGGGTGTGGGTGTGTGTGGGTGTG<br>GGTGTGGTGTGGGTGTGTGGGTGTGGTGTGTGGGTGTGGTGTGTGGGTGTGGTGTGTGGG<br>TGTGGGTGTGTGGGTGTGGTGTGTGGGTGTGGGTGTGGTGTGGGTGTGTGGGTGTGGTGT<br>GTGTGGGTGTGGTGTGTGTGGGTGTGGTGTGTGGGTGTGTGGGTGTGGTGTGTGTGTGGG<br>TGTGTGGGTGTGGTGTGGGTGTGGGTGTGGGTGTGGTGTGTGGGTGTGGTGTGGGTGTGG
18-bp *tlc1-tm* ITS	TGGTGTGGTGTGGTGTGG
34-bp *tlc1-tm* ITS	GTGGTGTGGTGTGGTGTGGTGTGGTGTGGTGTGG
50-bp *tlc1-tm* ITS	TGGTGTGGTGTGTGGTGTGGTGTGGTGTGGTGTGGTGTGGTGTGGTGTGG
100-bp *tlc1-tm* ITS	GTGTGGTGTGGTGTGTGGTGTGGTGTGTGGTGTGGTGGTGTGGTGTGGTGTGTGTGGTGT<br>GTGTGGTGTGTGGTGTGTGGTGTGTGGTGTGTGGTGTGTG
200-bp *tlc1-tm* ITS	GTGTGTGGTGTGGTGTGGATTTGGTGTGTGTGGTGTGGTGTGGTGTGGTGTGTGGTGTGTG<br>GTGTGTGGTGGTGTGGTGTGTGGTGTGTGGTGTGGTGTGGTGTGGTGTGGTGTGTGGTGT<br>GGTGTGTGGTGTGGTGGTGTGGTGTGGTGTGTGTGGTGTGTGTGGTGTGTGGTGTGTGGT<br>GTGTGGTGTGTGGTGTGTG
300-bp *tlc1-tm* ITS	TGGTGTGGTGTGTGGTGTGGTGTGGTGTGGTGTGGTGTGGTGTGGTGTGGGTGGTGTGTG<br>GTGTGGTGTGGTGTGGTGTGTGTGGTGGTGTGGTGGTGGTGTGTGTGGTGTGGTGTGGAT<br>TTGGTGTGTGTGGTGTGGTGTGGTGTGGTGTGTGGTGTGTGGTGTGTGGTGGTGTGGTGTG<br>TGGTGTGTGGTGTGGTGTGGTGTGGTGTGGTGTGTGGTGTGGTGTGTGGTGTGGTGGTGT<br>GGTGTGGTGTGTGTGGTGTGTGTGGTGTGTGGTGTGTGGTGTGTGGTGTGTGGTGTGTG
300-bp lambda phage	CCTTGTGACTGATGCAACTGACTCAGCACGCGCCGCCAGCACGTCCGCCGGACAGGCTGCAT<br>CGTCAGCTCAGGAAGCGTCCTCCGGCGCAGAAGCGGCATCAGCAAAGGCCACTGAAGCGG<br>AAAAAAGTGCCGCAGCCGCAGAGTCCTCAAAAAACGCGGCGGCCACCAGTGCCGGTGCGG<br>CGAAAACGTCAGAAACGAATGCTGCAGCGTCACAACAATCAGCCGCCACGTCTGCCTCCACC<br>GCGGCCACGAAAGCGTCAGAGGCCGCCACTTCAGCACGAGATGCGGTGGCCTCAAA
50-bp (TG)$_n$	TGTGTGTGTGTGTGTGTGTGTGTGTGTGTGTGTGTGTGTGTGTGTGTGTG
50-bp TTGGGG$_n$	GGGGTTGGGGTTGGGGTTGGGGTTGGGGTTGGGGTTGGGGTTGGGGTTGG
50-bp GGGGCC$_n$	GGGGCCGGGGCCGGGGCCGGGGCCGGGGCCGGGGCCGGGGCCGGGGCCGG
50-bp GGCCCC$_n$	GGCCCCGGCCCCGGCCCCGGCCCCGGCCCCGGCCCCGGCCCCGGCCCCGG
50-bp CCCTCT$_n$	CCCTCTCCCTCTCCCTCTCCCTCTCCCTCTCCCTCTCCCTCTCCCTCTCC
50-bp AGAGGG$_n$	AGAGGGAGAGGGAGAGGGAGAGGGAGAGGGAGAGGGAGAGGGAGAGGGAG
50-bp reverse ITS	CACACCCACACCCACACACACCACACCCACACACCCACACCACACCCACA
100-bp reverse ITS	CCACACCCACACCACACCCACACACCACACCCACACCCACACCCACACCACACCCACACACCC<br>ACACACACACCACACCCACACACCCACACACCACACC
300-bp reverse ITS	CCACACCCACACCACACCCACACACCACACCCACACCCACACCCACACCACACCCACACACCC<br>ACACACACACCACACCCACACACCCACACACCACACCCACACACACCACACCCACACACACCA<br>CACCCACACACCCACACCACACCCACACCCACACACCACACCCACACACCCACACCCACACAC<br>CACACCCACACACCACACCCACACACCACACCCACACACCCACACCACACCCACACCCACACA<br>CACCCACACCCACACACACCACACCCACACACCCACACCACACCCACA

Figure S1. GCR rate plotted as a function of wild-type ITS length. Data are the same as the wild-type data in Figure 1B, but plotted on a linear scale. Equations and R^2 values were determined using Microsoft Excel.

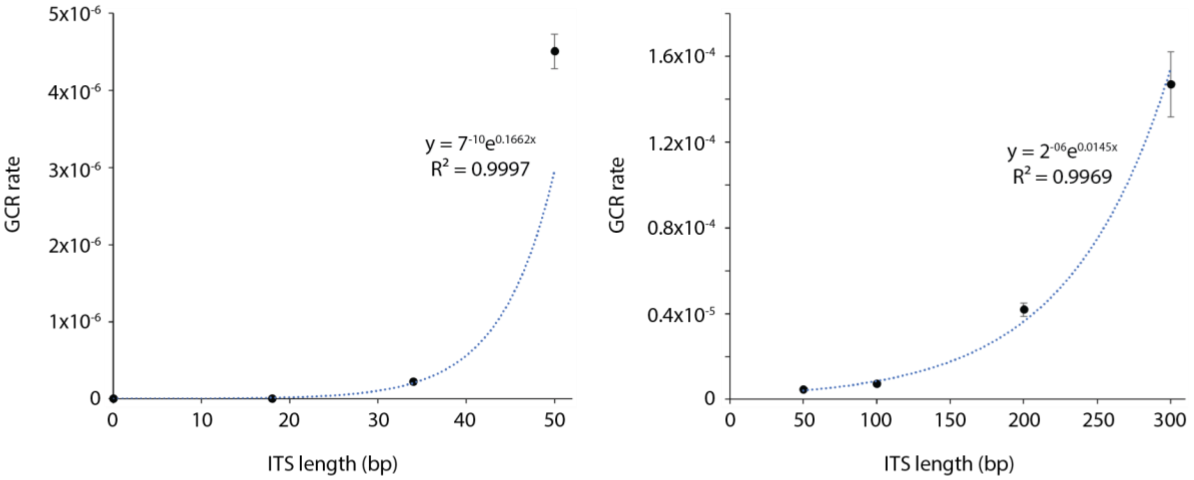

Figure S2. Resection is limited at a DNA end with 82 bp of telomeric sequence. An 82-bp ITS is located adjacent to an HO cut site at the *ADH4* locus on chromosome VII. The HO endonuclease gene is under the control of a galactose-inducible promoter. Cleavage at the HO cut site creates a DNA end with 82 bp of telomeric sequence, which will serve as a seed sequence for the addition of a new telomere by telomerase. Telomere addition events are selected based on the loss of the *LYS2* gene. The newly added telomeres of 21 independent isolates were sequenced and compared to the sequence of the original 82-bp ITS. The length of the original ITS retained in each newly added telomere is plotted. Three isolates retained 79 bp, and 4 isolates retained 81 bp. The remaining 14 isolates retained all 82 bp.

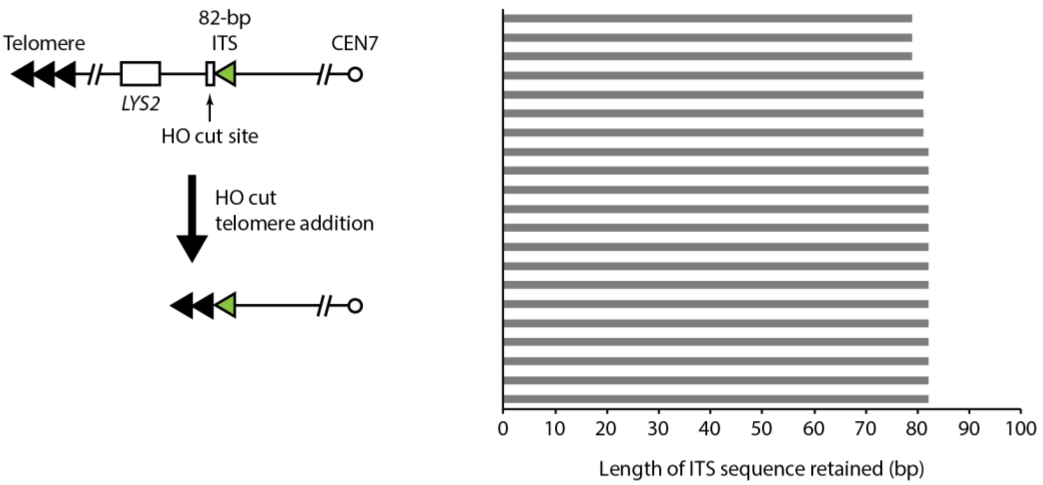

Figure S3. Rap1 protein levels in wild-type and *rpt4-150* strains. Wild-type and *rpt4-150* cells, harvested from logarithmically growing cultures, were fixed using TCA. Extracts from each sample were subjected to SDS-PAGE separation, followed by immunoblotting to detect Rap1 protein. Stain-Free imaging was used to assess the total protein input.

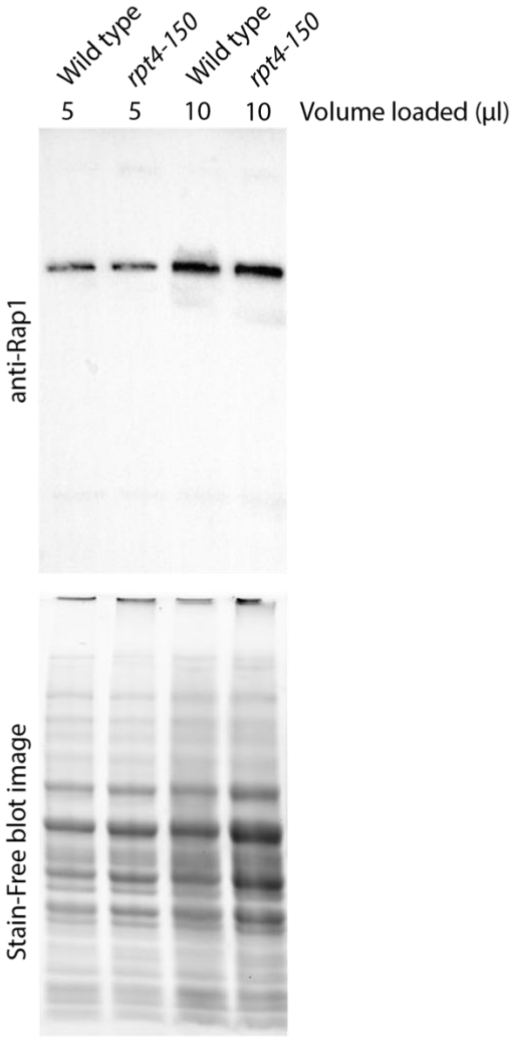

Figure S4. Deletion of *RAD10* does not eliminate GCR survivors that retain the entire ITS. (**A**) Model for how the Rad1-Rad10 endonuclease could function during de novo telomere addition. A break that occurs distal to the ITS will undergo 5'–3' end resection until a portion of the ITS becomes single stranded, which will then bind Cdc13 (not depicted). Cdc13 recruits telomerase, followed by Rad1-Rad10 cleavage of the 3' overhang/flap, allowing telomerase to extend the ITS into a de novo telomere. Deletion of *RAD1* or *RAD10* should reduce or eliminate GCR survivors that retain the entire ITS in the de novo telomere. (**B**) GCR survivors were isolated by growing *rad10Δ* strain with a 50-bp ITS on agar plates containing canavanine and 5-FOA. Nineteen independent isolates were analyzed. In all isolates, the presence of a de novo telomere added at the ITS site was confirmed by PCR and sequencing. The length of the original ITS retained for each de novo telomere is plotted. Nine out of 19 retain the entire ITS, including three isolates that also retained up to 14 bp of sequence downstream of the ITS (indicated by asterisks), contrary to what the model predicts.

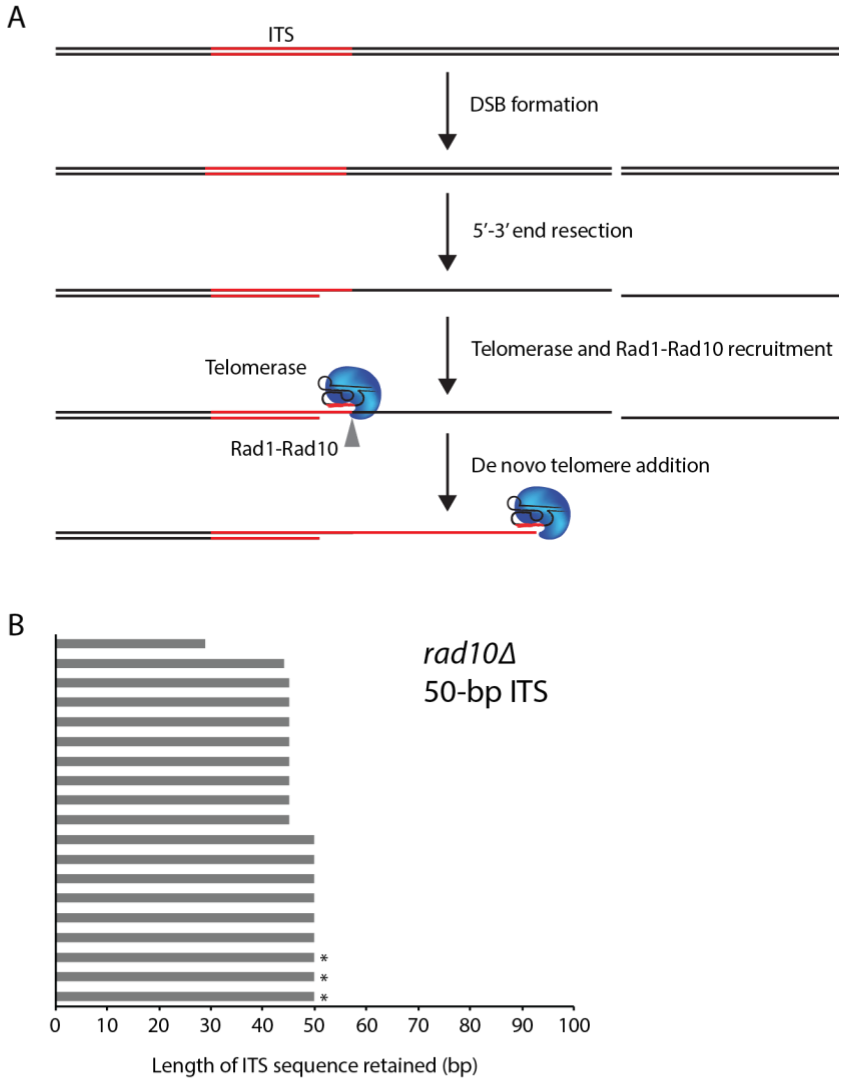

Figure S5. Genetic interaction profile similarity subnetwork for genes that suppress ITS-induced GCR rate. The network was generated using TheCellMap.org (3). Nodes (representing deletions of nonessential genes or temperature-sensitive alleles of essential genes) sharing similar genetic interaction profiles (PCC > 0.2) are connected by an edge in the network. Genes sharing similar genetic interaction profiles map closer to each other. The subnetwork was annotated using Spatial Analysis of Functional Enrichment (SAFE; 4).

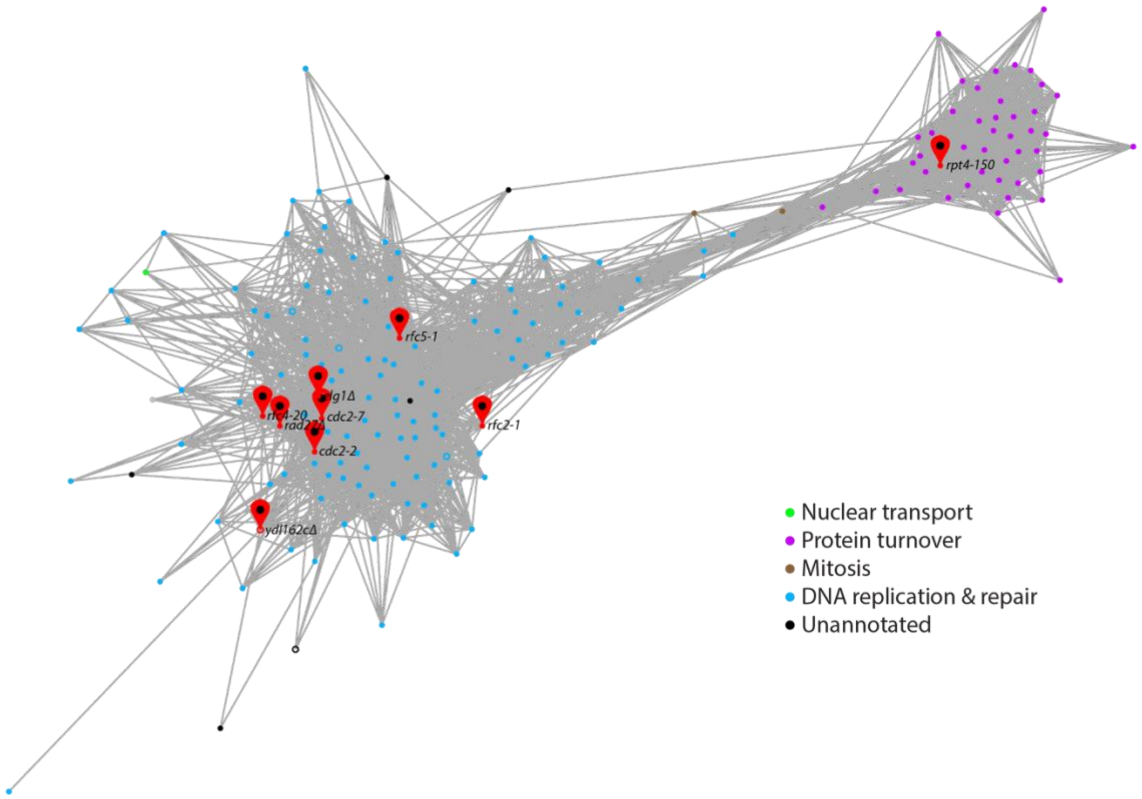

References

1. C. D. Putnam *et al.*, Bioinformatic identification of genes suppressing genome instability. *Proc Natl Acad Sci U S A* **109**, E3251-3259 (2012).
2. J. Strecker *et al.*, A sharp Pif1-dependent threshold separates DNA double-strand breaks from critically short telomeres. *eLife* **6**, e23783 (2017).
3. M. Usaj *et al.*, TheCellMap.org: A Web-Accessible Database for Visualizing and Mining the Global Yeast Genetic Interaction Network. *G3 (Bethesda)* **7**, 1539-1549 (2017).
4. A. Baryshnikova, Systematic Functional Annotation and Visualization of Biological Networks. *Cell Syst* **2**, 412-421 (2016).
